# Morphological, biophysical and synaptic properties of glutamatergic neurons of the mouse spinal dorsal horn

**DOI:** 10.1113/jphysiol.2013.264937

**Published:** 2014-01-08

**Authors:** Pradeep Punnakkal, Carolin Schoultz, Karen Haenraets, Hendrik Wildner, Hanns Ulrich Zeilhofer

**Affiliations:** 1 Institute of Pharmacology and Toxicology, University of Zurich, Winterthurerstrasse 190CH-8057, Zurich, Switzerland; 2 Institute of Pharmaceutical Sciences, Swiss Federal Institute of Technology (ETH) Zurich, Wolfgang Pauli-Strasse 10CH-8093, Zurich, Switzerland

## Abstract

Interneurons of the spinal dorsal horn are central to somatosensory and nociceptive processing. A mechanistic understanding of their function depends on profound knowledge of their intrinsic properties and their integration into dorsal horn circuits. Here, we have used BAC transgenic mice expressing enhanced green fluorescent protein (eGFP) under the control of the vesicular glutamate transporter (vGluT2) gene (vGluT2::eGFP mice) to perform a detailed electrophysiological and morphological characterisation of excitatory dorsal horn neurons, and to compare their properties to those of GABAergic (Gad67::eGFP tagged) and glycinergic (GlyT2::eGFP tagged) neurons. vGluT2::eGFP was detected in about one-third of all excitatory dorsal horn neurons and, as demonstrated by the co-expression of vGluT2::eGFP with different markers of subtypes of glutamatergic neurons, probably labelled a representative fraction of these neurons. Three types of dendritic tree morphologies (vertical, central, and radial), but no islet cell-type morphology, were identified in vGluT2::eGFP neurons. vGluT2::eGFP neurons had more depolarised action potential thresholds and longer action potential durations than inhibitory neurons, while no significant differences were found for the resting membrane potential, input resistance, cell capacitance and after-hyperpolarisation. Delayed firing and single action potential firing were the single most prevalent firing patterns in vGluT2::eGFP neurons of the superficial and deep dorsal horn, respectively. By contrast, tonic firing prevailed in inhibitory interneurons of the dorsal horn. Capsaicin-induced synaptic inputs were detected in about half of the excitatory and inhibitory neurons, and occurred more frequently in superficial than in deep dorsal horn neurons. Primary afferent-evoked (polysynaptic) inhibitory inputs were found in the majority of glutamatergic and glycinergic neurons, but only in less than half of the GABAergic population. Excitatory dorsal horn neurons thus differ from their inhibitory counterparts in several biophysical properties and possibly also in their integration into the local neuronal circuitry.

## Introduction

The spinal dorsal horn serves as the first relay station for sensory and nociceptive signals reaching the CNS from the periphery. Nociceptive (high-threshold) afferent fibres terminate mainly in its superficial layers (laminae I and II), while low-threshold mechanosensitive afferent fibres preferentially innervate the deep dorsal horn (laminae III–V). In both the superficial and the deep dorsal horn, more than 90% of the neurons are local interneurons. The proper functioning of these interneurons is an indispensable prerequisite for adequate perception of sensory stimuli in terms of quality, intensity and localisation (Graham *et al*. 2007; Todd, 2010; Zeilhofer *et al*. 2012a). A large body of evidence indicates that typical symptoms of chronic pain such as the increased sensitivity to noxious stimuli (hyperalgesia) and the painful perception of input from non-nociceptive fibres (allodynia) are at least partially due to dysfunctions of dorsal horn interneurons (Zeilhofer *et al*. 2012a).

A comprehensive mechanistic understanding of the role of these interneurons in the physiological processing of somatosensory and nociceptive signals and their malfunctioning in pathological pain states depends on a detailed knowledge of their biophysical properties and their integration in dorsal horn neuronal circuits. Most studies have so far focused on inhibitory interneurons. However, excitatory dorsal horn interneurons out-number their inhibitory counterparts by a factor of about two (Todd & Spike, 1993), and have recently been shown to be particularly important for supraspinally mediated pain behaviours (Wang *et al*. 2013).

Most previous electrophysiological studies addressing properties of defined subtypes of dorsal horn interneurons have relied on *post hoc* identification of neurons through neurochemical markers (Todd *et al*. 2003; Maxwell *et al*. 2007; Schneider & Walker, 2007; Yasaka *et al*. 2010; Polgár *et al*. 2013) or on simultaneous recordings of synaptically connected pairs of neurons (Lu & Perl, 2003, 2005). A more recently developed and in general more efficient approach is the use of reporter mice that express fluorescent proteins in defined neuronal subpopulations. Mice expressing enhanced green fluorescent protein (eGFP) in GABAergic neurons under the transcriptional control of the Gad67 or Gad65 gene, or in glycinergic neurons under the control of the GlyT2 (*Slc6a5*) gene have been successfully used to characterise dorsal horn inhibitory interneurons (Heinke *et al*. 2004; Zeilhofer *et al*. 2005; Gassner *et al*. 2009; Labrakakis *et al*. 2009; Cui *et al*. 2011). Corresponding marker genes for glutamatergic neurons belong to the family of vesicular glutamate transporters, which comprises three members, designated vGluT1 to vGluT3 (Chaudhry *et al*. 2008). The great majority of excitatory dorsal horn neurons express vGluT2 (*Slc17a6*), making this gene possibly well-suited as a marker gene for dorsal horn excitatory neurons (Oliveira *et al*. 2003; Todd *et al*. 2003; Alvarez *et al*. 2004). In the present study, we used a bacterial artificial chromosome (BAC) transgenic mouse line, which expresses eGFP under the transcriptional control of the vGluT2 gene, to perform targeted recordings from this interneuron population and to compare their intrinsic biophysical properties and their synaptic connections with those of GABAergic and glycinergic interneurons in Gad67::eGFP and GlyT2::eGFP transgenic mice.

## Methods

### Ethical approval

Permission for all animal experiments has been obtained from the Veterinäramt des Kantons Zürich (permissions 75/2010 and 86/2013). All experiments were carried out according to the guidelines laid down by the University of Zurich, and conform to the principles of UK regulations, as described in Drummond (2009).

### Mice

Experiments were carried out in three lines of genetically modified mice expressing eGFP either in glutamatergic neurons (vGluT2::eGFP mice; Gong *et al*. 2003), GABAergic neurons (Gad67::eGFP mice; Tamamaki *et al*. 2003), or glycinergic neurons (GlyT2::eGFP mice; Zeilhofer *et al*. 2005). Gad67::eGFP mice expressed eGFP from a targeted ‘knock-in’ insertion of eGFP into the Gad67 gene, while vGluT2::eGFP and GlyT2::eGFP mice expressed eGFP from a BAC transgene. Gad67::eGFP mice and GlyT2::eGFP mice have been previously described in detail (Tamamaki *et al*. 2003; Zeilhofer *et al*. 2005). vGluT2::eGFP mice [Tg(Slc17a6-EGFP)FY115 Gsat/MmucdI; BAC clone RP23–84M15] generated by the Gensat project (http://www.gensat.org) were obtained from the MMRRC (http://www.mmrrc.org). All mice had been backcrossed to the C57BL/6J background for at least 10 generations and were maintained on this background in a hemizygous/heterozygous state for the entire duration of this study. All three mouse lines have previously been used successfully by our group in studies on spinal dorsal horn neurons (Zeilhofer *et al*. 2005; Paul *et al*. 2012). All experiments were performed in mice between postnatal day P17 and P28 with exception of the experiments performed in parasagittal sections, which were done in 12- to 17-day-old mice.

### Immunohistochemistry

To study the dorsal horn eGFP expression pattern in the three transgenic mouse lines and to analyse the co-expression of vGluT2::eGFP with different interneuron markers, immunohistochemistry was performed on transverse sections of the lumbar mouse spinal cord. Mice were deeply anaesthetised with intraperitoneally injected pentobarbital followed by transcardiac perfusion of PBS and 30 ml ice-cold 4% paraformaldehyde (PFA) in PBS. After perfusion, the spinal cord was immediately isolated, postfixed in 4% PFA in PBS for 1–2 h and washed in PBS. The tissue was cryoprotected with 25% sucrose in PBS, embedded in Neg-50 frozen section medium (Thermo Scientific), frozen at −80°C, and cut into 25 *μ*m cryosections using a HYRAX C60 Cryostat (Carl Zeiss, Oberkochen, Germany). Sections were immediately mounted onto Superfrost Plus microscope slides (Thermo Scientific, Zurich, Switzerland) and stored at −80°C prior to use. The embedding medium was dissolved in PBS before sections were blocked in 0.1% Triton X-100/10% normal donkey serum (NDS, AbD Serotec, Kidlington, UK) in PBS. After overnight incubation with primary antibodies in blocking solution at 4°C, sections were washed and subsequently treated with secondary antibodies in blocking solution for 30–60 min at room temperature. After extensive washing in PBS followed by washing in 0.1× PBS, sections were covered in fluorescence mounting medium (Dako, Glostrup, Denmark). Coverslips were sealed with nail polish.

For diaminobenzidine (DAB) dependent staining, sections mounted on superfrost slides were incubated overnight at 4°C with a primary rabbit anti-GFP antibody in Tris buffer, pH 7.4, containing 2% normal goat serum (NGS, Biological Industries, Kibbutz Beit Haemek, Israel) and 0.2% Triton X-100. Sections were then washed and incubated for 30 min at room temperature with biotinylated secondary antibody (1:300; Jackson ImmunoResearch, Suffolk, UK), followed by incubation in avidin–biotin complex (1:100 in Tris buffer, Vectastain Elite Kit; Vector, Laboratories, Burlingame, CA, USA), for 30 min, washed again, and finally visualised with DAB tetrahydrochloride (Sigma-Aldrich, St Louis, MO, USA) in Tris buffer (pH 7.7) containing 0.015% hydrogen peroxide. The colour reaction was stopped after 5–15 min with ice-cold PBS. Sections were covered in mounting medium (Dako).

The following antibodies were used: rabbit anti-GFP (1:1000 or 1:3000; Molecular Probes Inc., Eugene, OR, USA), sheep anti-GFP (1:1000; AbD Serotec, Kidlington, UK), mouse anti-NeuN (1:500; Millipore, Billerica, MA, USA), rabbit anti-Pax2 (1:400; Invitrogen, Carlsbad, USA), mouse anti-protein kinase C*γ* (anti-PKC*γ*, 1:1000; BD Biosciences, Franklin Lakes, NJ, USA), rabbit anti-PKC*γ* (1:1000; Santa Cruz, Dallas, TX, USA), rabbit anti-c-Maf (1:10,000; gift from Dr Carmen Birchmeier, MDC Berlin), mouse anti-calbindin D-28k (1:5000; Swant, Marly, Switzerland), rabbit anti-substance P receptor (NK1 receptor, 1:5000; Sigma-Aldrich) and cyanine 3 (Cy3)-, Alexa Fluor 488-, DyLight 488-, 647- and 649-conjugated donkey secondary antibodies (Dianova, Hamburg, Germany).

### Dendritic tree morphology

To analyse the dendritic tree morphology of vGluT2::eGFP neurons, targeted whole-cell recordings were performed in 300 *μ*m thick parasagittal slices with recording pipettes filled with internal solution containing 0.5% biocytin (Sigma-Aldrich). After reaching the whole-cell configuration, the pattern of action potential firing was determined as described below and recordings were maintained for at least 15 min before the pipette was carefully removed from the recorded neuron. Slices were fixed overnight in 4% PFA, containing 15% picric acid, and subsequently stored in 10% saccharose–0.05% NaN_3_ in PBS until further analysis. Without further sectioning, free-floating slices were washed briefly in PBS and permeabilised extensively in 1% Triton X-100–10% normal NGS in PBS for up to 5 h at room temperature. Subsequently, slices were incubated at 4°C for 2 days in polyclonal rabbit anti-GFP antibody solution (1:2000; Synaptic Systems, Göttingen, Germany) to verify eGFP expression of the recorded cell, and in Alexa Fluor 488-conjugated streptavidin (1:150; Dianova) to label the biocytin-filled neuron. Next, sections were washed, permeabilised again and incubated in Cy3-conjugated goat anti-rabbit secondary antibody (1:500; Dianova) for 2 h at room temperature and briefly in 4′,6-diamidino-2-phenylindole (DAPI; 1:10,000). All antibodies were diluted in 10% NGS–0.1% Triton X-100 in PBS. Sections were air-dried and mounted on gelatin-coated glass slides (Thermo Scientific) in fluorescence mounting medium (Dako).

### Image analysis

Fluorescent images were acquired on a Zeiss LSM710 Pascal confocal microscope using a 0.8 NA × 20 Plan-apochromat objective or a 1.3 NA × 40 EC Plan-Neofluar oil-immersion objective and the ZEN2012 software (Carl Zeiss). For colocalisation studies, confocal settings (confocal aperture, laser power, gain, offset, pixel dwell and pixel size) were identical for all scans of the same staining. To generate images displaying the gross distribution of dorsal horn eGFP expression, maximum intensity projections of stacks were made. Whenever applicable, contrast, illumination, and false colours were adjusted in ImageJ or Adobe Photoshop (Adobe Systems, Dublin, Ireland). Quantification of co-localisation was performed on three single optical sections of three mice each in ImageJ using the Cell Counter Plugin.

### Electrophysiology

Transverse 250–350 *μ*m thick lumbar spinal cord slices with short dorsal roots attached were prepared as described previously (Kato *et al*. 2012). After transfer to the recording chamber, slices were continuously superfused with oxygenated (95% O_2_–5% CO_2_) extracellular solution containing (in mm): 120 NaCl, 26 NaHCO_3_, 1.25 NaH_2_PO_4_, 2.5 KCl, 2 CaCl_2_, 1 MgCl_2_, 5 Hepes, 10 glucose (pH 7.35, 305–315 mosmol l^−1^). Whole-cell patch-clamp recordings were made at room temperature from dorsal horn neurons visually identified with the infrared gradient contrast technique. Recording pipettes had resistances of 3–6 MΩ and were filled with internal solution containing (in mm): 130 potassium gluconate, 5 NaCl, 1 EGTA, 5 Mg-ATP, 0.5 Na-GTP, 10 Hepes (pH 7.35, adjusted with KOH, 290–300 mosmol l^−1^). For experiments involving the recording of postsynaptic currents (EPSCs and IPSCs) K^+^ was replaced by equimolar Cs^+^. Resting membrane potential, cell capacitance, input resistance and action potential firing were studied in the current clamp mode. Input resistance was determined through injection of a hyperpolarising current for 500 or 2000 ms, for transverse and parasagittal slices respectively. Action potential firing was evoked by repeated depolarising current injections of 500 ms duration with step-wise increasing amplitudes (−20 to +240 pA). Primary afferent-evoked postsynaptic currents were elicited at a frequency of 1/15 s by electrical stimulation of the dorsal root (stimulation intensity 15–70 V, stimulus width 300–500 *μ*s) and recorded either at a holding potential of −80 mV (EPSCs) or at 0 mV (IPSCs). For electrical dorsal root stimulation, either a bipolar electrode with the poles placed on both sides of the dorsal root or a wide bore glass (suction) electrode with the distal end of the dorsal root held inside was used. To avoid undesired direct stimulation of intrinsic dorsal horn neurons, suction and reference electrodes were placed on the same (dorsal) side of the slice preparation. The presence of capsaicin-sensitive input to the recorded neuron was tested by superfusing the slice with extracellular solution containing 1 *μ*m capsaicin (dissolved in 0.1% DMSO).

## Results

The vGluT2::eGFP transgenic mouse line used in this study has not been characterised in detail before. We therefore began our study with a characterisation of the gross distribution of eGFP-tagged neurons in the lumbar dorsal horn of vGluT2::eGFP transgenic mice (Fig. 1*A*). Transverse sections of PFA-fixed spinal cord tissue were prepared from three mice and endogenous eGFP fluorescence was enhanced through staining with anti-GFP antisera. eGFP-positive somata were rather densely packed in the superficial dorsal horn (laminae I and II) and around the central canal (region X according to Rexed, 1952; Fig. 1*Aa* and *b*). Additional eGFP-positive somata were found in the deep dorsal horn and a few scattered eGFP-positive somata were also distributed in the ventral horn (Fig. 1*Ac*). Within the spinal grey matter, the gross distribution of vGluT2::eGFP-positive neuropil largely paralleled that of eGFP-positive somata, but intense neuropil staining was also present in dorsolateral funiculus (Fig. 1*A* and *Ba*), which contains the axons of contralateral lamina I projection neurons (McMahon & Wall, 1985). In Gad67::eGFP mice, eGFP-positive somata and neuropil were most abundant in lamina II and thus exhibited a distribution similar to that of eGFP in the vGluT2::eGFP mice (Fig. 1*Bb*). The laminar distribution of GlyT2::eGFP neurons was different from that of vGluT2::eGFP and Gad67::eGFP neurons, in that GlyT2::eGFP-positive neurons were most abundant in lamina III and deeper, but largely absent from lamina II and only sparsely present in lamina I. However, eGFP-positive neuropil extended clearly into lamina I and II (Fig. 1*Bc*).

**Figure 1:**
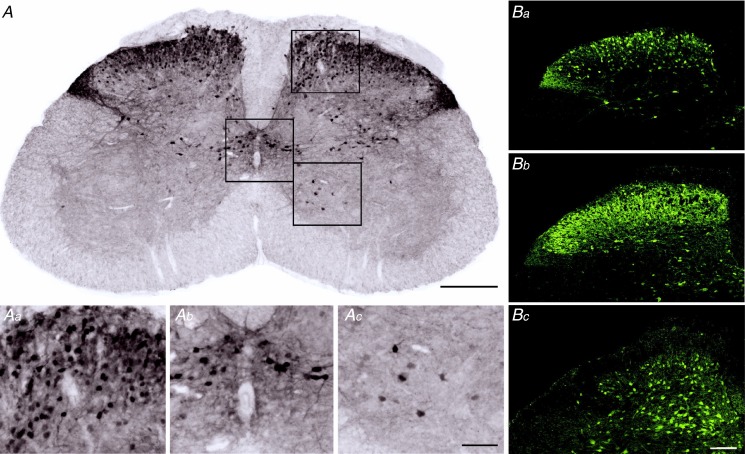
*A*, immunoperoxidase staining of vGluT2::eGFP in the lumbar spinal cord. Scale bar, 300 *μ*m. *Aa–c*, higher magnifications of selected areas (*a*, dorsal horn; *b*, around the central canal; *c*, ventral horn). Magnified areas are indicated as squares in *A*. Scale bar, 100 *μ*m. *B*, eGFP expression the dorsal horn of vGluT2::eGFP (*a*), Gad67::eGFP mice (*b*), and GlyT2::eGFP mice (*c*). Scale bar, 100 *μ*m.

We next performed a quantitative analysis of vGluT2::eGFP neurons in the dorsal horn. Because a general neurochemical marker reliably labelling the somata of all dorsal horn glutamatergic neurons has not been available, we identified excitatory neurons indirectly through the absence of staining for Pax2, and positive labelling with the pan-neuronal marker NeuN (Mullen *et al*. 1992; Fig. 2*A*). It has previously been shown that Pax2 is expressed in the vast majority of spinal inhibitory interneurons during mouse development (Maricich & Herrup, 1999; Cheng *et al*. 2004), but it was not known whether Pax2 remains expressed in the adult. We found that Pax2 expression is maintained into adulthood in both Gad67::eGFP neurons (93 ± 2%) and in GlyT2::eGFP neurons (92 ± 4%), demonstrating that Pax2 constitutes a suitable marker for dorsal horn inhibitory interneurons. Because of the presence of a dorso-ventral gradient in the density of vGluT2::eGFP neurons (see panels *A* and *Ba* of Fig. 1), subsequent analyses were made separately for the superficial dorsal horn (laminae I and II) and the deep dorsal horn (lamina III and deeper). Immunostaining against PKC*γ*, which labels the innermost part of lamina II (Martin *et al*. 1999; Polgár *et al*. 1999), was used to delineate the border between lamina IIi and III. A total of nine sections from three mice were analysed. We counted 1693 Pax2-negative (but NeuN-positive) superficial dorsal horn somata of which 753 (39.9 ± 23.1%, mean ± SEM, in *n* = 3 mice) were eGFP positive. In the deep dorsal horn, we counted 1997 Pax2-negative (NeuN-positive) neurons of which 476 were eGFP positive (23.9 ± 8.9%). Among a total of 1232 vGluT2::eGFP-positive neurons only three showed apparent expression of Pax2, indicating that eGFP expression in vGluT2::eGFP mice was virtually confined to excitatory glutamatergic neurons.

**Figure 2:**
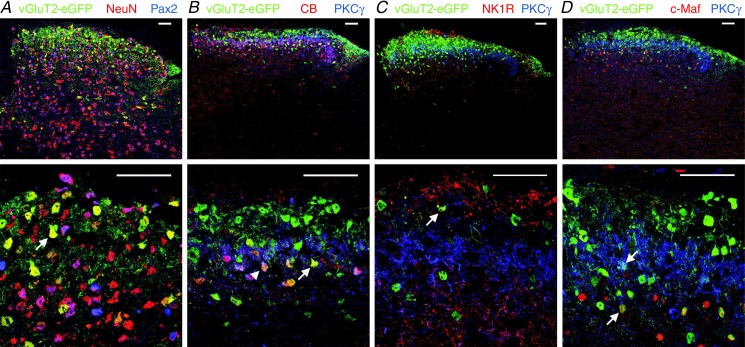
*A*, co-staining of eGFP in dorsal horn sections of vGluT2::eGFP BAC transgenic mice with the pan-neuronal marker NeuN and the inhibitory interneuron marker Pax2. Arrow indicates a neuron co-expressing vGluT2::eGFP and NeuN. *B–D*, co-stainings of eGFP and of four established markers of subpopulations of dorsal horn excitatory neurons (CB, PKC*γ*, NK1R, and c-Maf). Examples of neurons co-expressing eGFP with CB (*B*), NK1R (*C*), and c-Maf (*D*) are indicated by upward arrows. The downward arrow (in *D*) indicates a neuron co-expressing eGFP and PKC*γ*. The arrowhead in *B* marks a triple-positive (eGFP/CB/PKC*γ* expressing) neuron. PKC*γ* staining was used in *B–D* to delineate the border of lamina IIi and lamina III. Top row, whole dorsal horn. Bottom row, laminae I–III at higher magnification. All images depict single optical sections. Scale bars, 50 *μ*m.

Our observation that vGluT2::eGFP was expressed only in 20–40% of all presumed excitatory neurons raised the possibility that eGFP was expressed only in a specific subpopulation of dorsal horn excitatory neurons. We therefore went on to investigate the presence of eGFP in subsets of excitatory neurons expressing calbindin-D 28k (CB; Antal *et al*. 1991), NK1 receptors (Al-Khater *et al*. 2008), PKC*γ* (Polgár *et al*. 1999), or the transcription factor c-Maf (Hu *et al*. 2012) (Fig. 2*B–D*). The vast majority of NK1 receptor-positive neurons were located in the superficial dorsal horn (168 out of 175) and more than 80% of all PKC*γ*-positive neurons were within lamina II (see also Moussaoui *et al*. 1992; Martin *et al*. 1999; Polgár *et al*. 1999). Consistent with a previous report (Hu *et al*. 2012), c-Maf-positive neurons were more abundant in the deep dorsal horn and calbindin D 28k-positive neurons were more numerous in the superficial dorsal horn (compare also Yamamoto *et al*. 1989 and Celio, 1990). Within the four subpopulations of excitatory dorsal horn neurons, between 21.7 and 32.8% of neurons also expressed vGluT2::eGFP (Table 1). These percentages were not significantly different from that determined for the whole population of Pax2-negative neurons (33.3%; one-way ANOVA *F*(4,10) = 0.67; *P* = 0.63). vGluT2::eGFP-positive neurons thus appear to constitute a representative portion of all dorsal horn excitatory neurons. Consistent with this notion, PKC*γ* or NK1 receptor expression, which both define rather small subpopulations of dorsal horn excitatory neurons, were detected only in 2.0–6.5% of vGluT2::eGFP neurons (Table 1).

**Table 1:** Analyses of co-expression with vGluT2::eGFP of markers (calbindin, NK1 receptor, PKC*γ*, and cMaf) of subpopulations of excitatory neurons

	Subpopulation marker				
	Calbindin D-28k	NK1 receptor	PKC*γ*	c-Maf	Pax2^−^ / NeuN^+^
Double labelled/ marker positive	134/409 (32.8%; 19.2–42.2%)	38/175 (21.7%; 16.3–29.9%)	34/134 (25.4%; 20.6–30.3%)	239/868 (27.5%; 13.1–33.3%)	1229/3690 (33.3%; 15.7–48.6%)
Double labelled/vGluT2::eGFP	134/901 (14.9%; 14.9%)	38/1178 (3.2%; 2.0–5.0%)	34/901 (3.8%; 2.9–6.5%)	239/1273 (18.8%; 9.1–24.0%)	1229/1232 (99.8%; 99.5–99.9%)

Data show absolute numbers, percentage and percentage range from three mice. The last column indicates the number of non-inhibitory (presumed excitatory) neurons expressing vGluT2::eGFP and the portion of presumed excitatory neurons among the vGluT2::eGFP-positive neurons. First row, no significant differences were found in the percentage of calbindin D-28k, NK1 receptor, PKC*γ*, c-Maf-positive or Pax2-negative neurons expressing vGluT2::eGFP (one-way ANOVA *F*(4,10) = 0.67; *P* = 0.63). Second row, percentage of calbindin D-28k, NK1 receptor, PKC*γ*, or cMaf-positive neurons among the vGluT2::eGFP neurons differed significantly between the four markers (one-way ANOVA (*F*(4,10) = 9.44, *P* < 0.01). Colocalisations of PKC*γ* with eGFP were determined from the sections also stained for calbindin.

Dorsal horn interneurons have also been classified according to the morphology of their dendritic trees. Most studies have focused on the superficial dorsal horn, where most reports distinguished islet cells, central cells, radial cells, and vertical cells (Grudt & Perl, 2002; for a recent review see Zeilhofer *et al*. 2012b). To investigate the morphology of the dendritic trees of vGluT2::eGFP neurons, we filled superficial dorsal horn neurons with biocytin during whole-cell recording for *post hoc* classification of their primary dendritic trees (Fig. 3). Because the vast majority of lamina II interneurons exhibit a predominant rostrocaudal spread of their dendrites, we performed these analyses on parasagittal slices in order to maintain the cells’ integrity as far as possible. For 27 cells, we obtained both dendritic tree morphology and firing behaviour. Twenty of these neurons could be classified as either vertical, central, or radial cells. Seven cells displayed a ‘central cell’ morphology having an average spread in the rostro-caudal direction of 145.9 ± 18.2 *μ*m and a limited dorso-ventral spread of 47.8 ± 6.5 *μ*m (Fig. 3*B*, Table 2). A further seven cells were classified as ‘radial cells’ and showed a more equal rostro-caudal to dorso-ventral spread compared to central cells (153.7 ± 23.6 and 93.8 ± 13.6 *μ*m, respectively), and six cells were termed ‘vertical cells’ as their dendrites predominantly extended into the ventral direction (average dorso-ventral spread 152.2 ± 19.9 *μ*m). The remaining seven cells had diverse morphological appearances and did not fit into any of the four categories. Importantly, none of the vGluT2::eGFP cells showed an islet cell morphology and none of our cells fulfilled the criteria of the medial–lateral cells described by Grudt & Perl (2002), i.e. dendritic spreads in the medio-lateral direction never exceed 60 *μ*m. To ensure that this result was not due to insufficient antibody penetration, we analysed the dorso-ventral spread in transverse sections (data not shown) and found that the average distance from the soma centre to the ventral end of the dendritic tree was always less than 130 *μ*m (*n* = 10).

**Table 2:** Values show the means ± SEM (in *μ*m) of measured primary dendritic spread

	Dendritic arbour dimensions (*μ*m)			
			Soma centre to	Soma centre to
	Rostro-caudal	Dorso-ventral	dorsal end	ventral end
Central cells (*n* = 7)	146 ± 18	48 ± 7	24 ± 4	24 ± 3
Radial cells (*n* = 7)	154 ± 24	**94** **± 14**	47 ± 8	45 ± 7
Vertical cells (*n* = 6*)*	239 ± 19	152 ± 20	36 ± 5	**115** **± 17**

Dimensions that helped to classify a particular morphological cell type are in bold. The unclassified cells are not shown here. Shrinkage during histological preparation was not taken into account.

**Figure 3:**
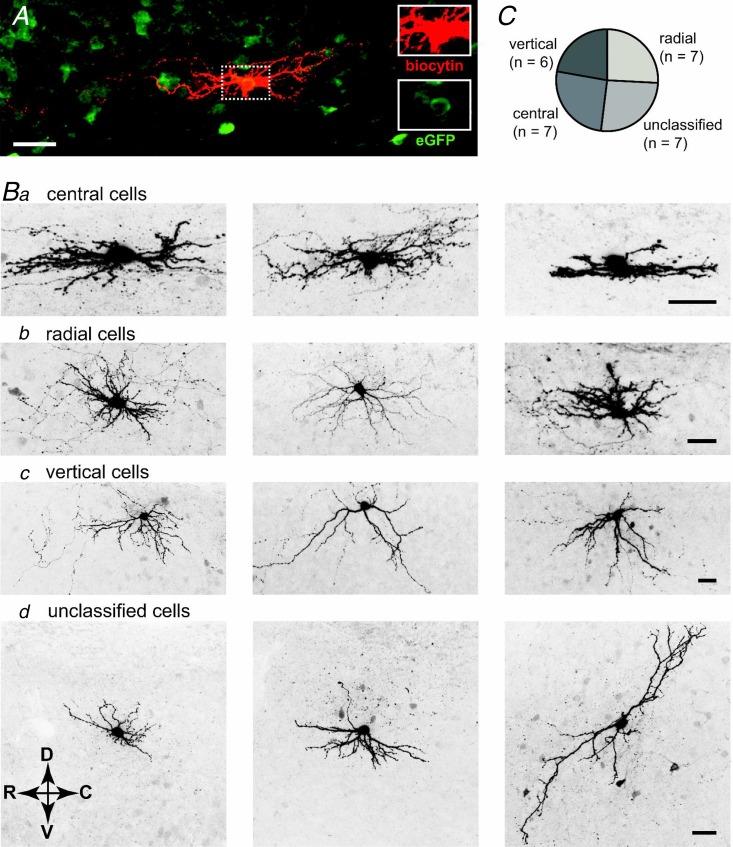
*A*, example of a central cell filled with biocytin and stained with fluorescently labelled antibodies. Insets on the right show intracellular biocytin (red) and presence of eGFP (green). *B*, confocal images showing the somata and primary dendritic trees of 12 dorsal horn vGluT2::eGFP cells. Fluorescent images were taken from 300 *μ*m parasagittal sections and acquired on a confocal microscope. Z-stacks of biocytin filled and streptavidine-Alexa Fluor 488 stained neurons consisted of 40–60 optical sections separated by 0.5 *μ*m steps. Three examples of each category are shown. Cells were classified as central cells (*a*), radial cells (*b*), vertical cells (*c*), and unclassified cells (*d*). Additional cell bodies became sometimes visible in the background due to detection of eGFP fluorescence remaining after fixation. Arrows indicate orientation of the parasagittal sections. *C*, prevalence of the different morphological subtypes (*n* = 27). All scale bars, 30 *μ*m.

We then characterised the biophysical properties of vGluT2::eGFP neurons and compared them with those of Gad67::eGFP and GlyT2::eGFP neurons (Table 3). A total of 113 neurons were recorded. Resting membrane potential (*V*_rest_), cell capacitance (*C*_cell_) and input resistance (*R*_input_) of vGluT2::eGFP neurons (*n* = 41) were not significantly different from those of Gad67::eGFP (*n* = 41) and GlyT2::eGFP neurons (*n* = 31). Significant differences were, however, obtained for the rheobase (minimum current amplitude that evoked at least one action potential), the action potential threshold, and the action potential width. vGluT2::eGFP neurons had a higher rheobase and more depolarised action potential thresholds indicating that they required stronger excitatory input for activation. Action potentials of vGluT2::eGFP neurons were shorter than those of Gad67::eGFP and GlyT2::eGFP neurons. Significant differences were also found between Gad67::eGFP and GlyT2::eGFP neurons. Gad67::eGFP neurons had a smaller rheobase and a less depolarised action potential threshold than GlyT2::eGFP neurons, indicating that they were more readily excited by depolarising synaptic input than GlyT2::eGFP neurons. Furthermore, action potentials in GlyT2::eGFP neurons were shorter than in Gad67::eGFP neurons.

**Table 3:** Passive and active biophysical properties of vGluT2::eGFP-positive dorsal horn neurons and of their inhibitory counterparts (Gad67::eGFP and GlyT2::eGFP neurons)

	*V*_rest_ (mV)	*C*_cell_ (pF)	*R*_input_ (MΩ)	Rheobase (pA)	AP threshold (mV)	AP width^1^ (ms)	After-hyper-polarisation (mV)
vGluT2 *(n *= 41*)*	−64.2 ± 1.4	38 ± 5.5	1038 ± 108	54.6 ± 5.0	−27.9 ± 1.0	3.52 ± 0.15	−21.6 ± 0.9
Sign *vs*. Gad67	—	—	—	***	***	(*P* = 0.052)	—
Sign *vs*. GlyT2	—	—	—	***	***	***	—
Gad67 *(n = 41)*	−62.4 ± 1.7	36 ± 4.7	1157 ± 97	18.1 ± 1.9	−39.5 ± 0.7	3.01 ± 0.18	−21.6 ± 1.1
Sign *vs*. GlyT2	—	—	—	(*P* = 0.051)	***	***	—
GlyT2 *(n = 31)*	−66.8 ± 1.9	36 ± 6.7	1260 ± 172	31.9 ± 4.2	−33.4 ± 1.4	2.09 ± 0.10	−21.8 ± 0.8

1 Determined at the AP base. No separate analyses were made for neurons of the superficial or deep dorsal horn. Values are means ± SEM.

*** *P* ≤ 0.001;

** *P* ≤ 0.01. ANOVA followed by Bonferroni *post hoc* test. *F*(2,110) = 1.71 (resting membrane potential); 0.045 (cell capacitance); 0.76 (input resistance); 24.1 (rheobase); 34.5 (action potential (AP) threshold); 19.7 (action potential width); 28.3 (after-hyperpolarisation).

We next analysed the action potential firing patterns of vGluT2::, Gad67::, and GlyT2::eGFP neurons (Fig. 4). These analyses were again done separately for superficial and deep dorsal horn neurons. Changes in membrane voltage were recorded in the current-clamp mode of the patch-clamp technique. First, a hyperpolarising current of −20 pA was injected into the recorded cell for 500 ms to measure the input resistance of the recorded cell (bottom trace). Subsequently, depolarising current injections of the same duration with amplitudes increasing in steps of 20 pA were applied at intervals of 12 s. Figure 4*Aa–d* displays action potential firing patterns in vGluT2::eGFP neurons observed at the rheobase (middle trace) and after injection of a depolarising current that was sufficient to evoke a maximum number of action potentials (top trace). Action potential firing patterns were classified as single spike, delayed, phasic, and tonic firing based on the responses observed during a depolarisation that evoked a maximal number of action potentials. Single spiking neurons typically fired their action potential at the beginning of the depolarisation (Fig. 4*Aa*). No additional action potentials were evoked even with depolarising current injections of three times the threshold current amplitude. In neurons with delayed action potential firing the first action potential always occurred with a certain (>100 ms) delay. Suprathreshold depolarisations often evoked multiple action potentials occurring at irregular intervals (Fig. 4*Ab*). Neurons with phasic firing patterns produced two or more bursts of action potentials separated by silent periods of at least 100 ms. (Fig. 4*Ac*). Neurons exhibiting more than one burst of action potentials but with the first action potential occurring only after a delay were still classified as delayed firing neurons. Neurons with tonic action potential firing pattern fired already multiple action potentials at threshold depolarisations. With stronger current injections, action potentials occurred at high frequencies (up to 50 Hz), often with at least some degree of frequency adaptation (Fig. 4*Ad*).

**Figure 4:**
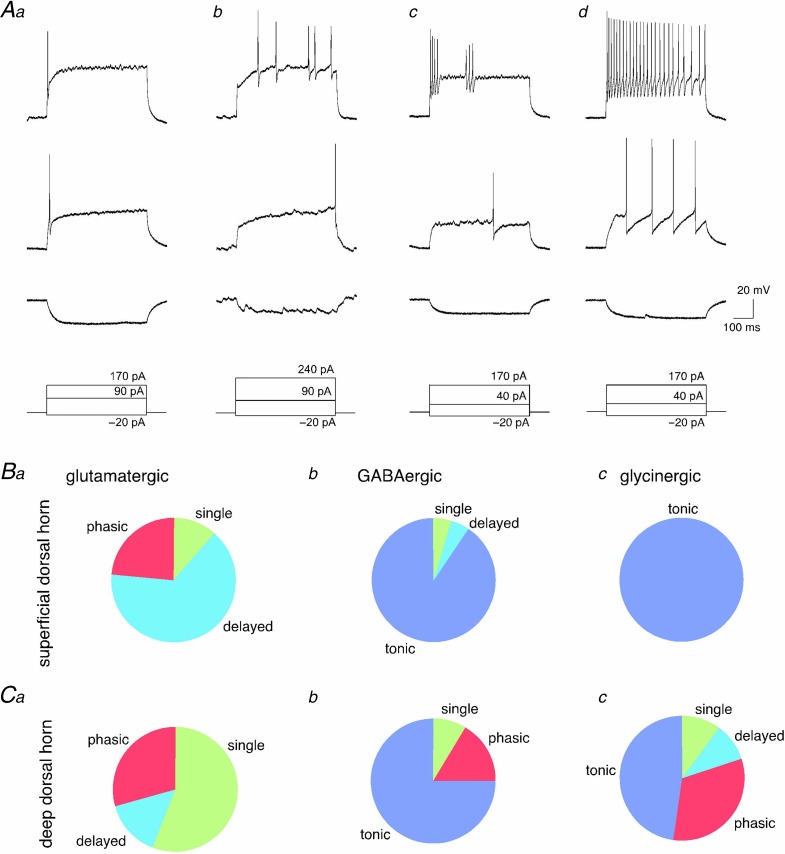
*A*, action potentials were evoked by depolarising current injections of different amplitudes. Each column represents voltage responses of a given neuron. Traces show voltage response to a hyperpolarising current injection (bottom), to the minimum current injection that was capable to elicit at least one action potential (rheobase, middle), and to a current injection evoking a maximum number of action potentials (top). Voltage recordings *Aa–Ac* were from a vGluT2::eGFP-expressing neuron. *Ad* is from a Gad67::eGFP-positive neuron. Four patterns of action potential firing could be distinguished (from left to right): single, delayed, phasic, and tonic action potential firing. *B* and *C*, prevalence of the four different action potential firing patterns in glutamatergic (vGluT2::eGFP-positive; *a*), GABAergic (Gad67::eGFP-positive, *b*), and glycinergic (GlyT2::eGFP-positive, *c*) neurons of the superficial and deep dorsal horn (*B* and *C*, respectively). Total numbers of vGluT2::eGFP, Gad67::eGFP and GlyT2::eGFP-positive cells were *n* = 29, 23, 6, and *n* = 7, 13, and 21, for neurons from the superficial and deep dorsal horn, respectively. For statistical comparisons see Results.

The most prevalent action potential firing pattern in superficial dorsal horn vGluT2::eGFP neurons was delayed firing (19 out of 29 neurons, 66%) followed by phasic (7 of 29, 24%), and single (3 of 29, 10%) action potential firing (Fig. 4*Ba*). No tonically firing neurons were detected among the vGluT2::eGFP neurons. The prevalence of the different action potential firing patterns in inhibitory neurons of the superficial dorsal horn was strikingly different from those of their excitatory counterparts (*χ*^2^(6) = 51.4; *P* < 0.001). Pair-wise comparisons revealed significant differences both between vGluT2::eGFP neurons and Gad67::eGFP neurons (*χ*^2^(3) = 45; *P* < 0.001) and between vGluT2::eGFP neurons and GlyT2::eGFP neurons (*χ*^2^(3) = 35; *P* < 0.001). The great majority of all inhibitory neurons exhibited a tonic firing pattern (21 of 23 Gad67::eGFP and 6 of 6 GlyT2::eGFP neurons) (Fig. 4*Bb* and *c*). The two non-tonically firing Gad67::eGFP neurons showed delayed action potential firing or fired a single action potential. Differences in the prevalence of the different firing patterns between the two groups of inhibitory neurons (Gad67::eGFP and GlyT2::eGFP) were insignificant (*χ*^2^(3) = 0.56; *P* = 0.76).

In the deep dorsal horn, differences in prevalence of the four firing patterns were smaller but still significant (*χ*^2^(6) = 16.0; *P* < 0.05; Fig. 4*Ca–c*). Pair-wise comparisons again indicated significant differences between vGluT2::eGFP neurons and Gad67::eGFP neurons as well as between vGluT2::eGFP neurons GlyT2::eGFP neurons (*χ*^2^(3) = 12.1; *P* < 0.01, and *χ*^2^(3) = 9.0; *P* < 0.05, respectively). The majority of vGluT2::eGFP neurons (4 of 7) fired only a single action potential even after strong depolarisation, while 2 of 7 cells showed phasic firing, and only a single cell had a delayed firing pattern (Fig. 4*Ca*). Tonic firing still dominated in inhibitory neurons (10 of 13 Gad67::eGFP and 10 of 21 GlyT2::eGFP neurons), but single, phasic and delayed firing patterns were more prevalent in inhibitory neurons of the deep than of the superficial dorsal horn (Fig. 4*Cb* and *c*).

For those neurons in which we had characterised the dendritic tree morphology (Fig. 3) we had also analysed the firing pattern. This allowed us to test whether the different firing behaviours correlated with particular dendritic tree morphologies. Our analysis revealed that all vertical neurons (*n* = 6) had a delayed firing phenotype, whereas no defined firing pattern could be assigned to central and radial cells. Of both the seven central and seven radial cells studied here, four showed a delayed firing, two a phasic and one a single-spiking firing pattern. The prevalence of the different firing patterns was similar to that obtained in transverse sections with about 65% delayed firing, 18% phasic, and 12% single spike firing neurons (compare Fig. 4).

We then tested whether biophysical properties differed between vGluT2::eGFP neurons with different firing patterns and compared these properties in addition to those of tonically firing Gad67::eGFP neurons (Fig. 5). No differences were found for the resting membrane potential (Fig. 5*A*) and the amplitude of the after-hyperpolarisation (Fig. 5*E*). However, action potential thresholds and rheobase differed significantly between neuron types (Fig. 5*B*,*C*). Single spike, delayed and phasic firing vGluT2::eGFP neurons had more depolarised action potential thresholds than tonic firing Gad67::eGFP neurons. Furthermore, delayed firing vGluT2::eGFP neurons had significantly more depolarised action potential thresholds than their single spiking cousins (one-way ANOVA F(3,69) = 29.4; *P* < 0.05; Fig. 5*B*). The rheobase of single spiking and delayed firing vGluT2::eGFP neurons was significantly larger than that of tonic Gad67::eGFP neurons (one-way ANOVA *F*(3,69) = 22.1; *P* < 0.001 for both types of neurons), and single spiking vGluT2::eGFP neurons had a significantly larger rheobase than phasic vGluT2::eGFP neurons (one-way ANOVA *F*(3,69) = 22.1; *P* < 0.01; Fig. 5*C*). Finally, a small yet statistically significant difference was detected in the action potential width between delayed vGluT2::eGFP neurons and tonic Gad67::eGFP neurons (one-way ANOVA *F*(3,69) = 4.28; *P* < 0.01; Fig. 5*D*).

**Figure 5:**
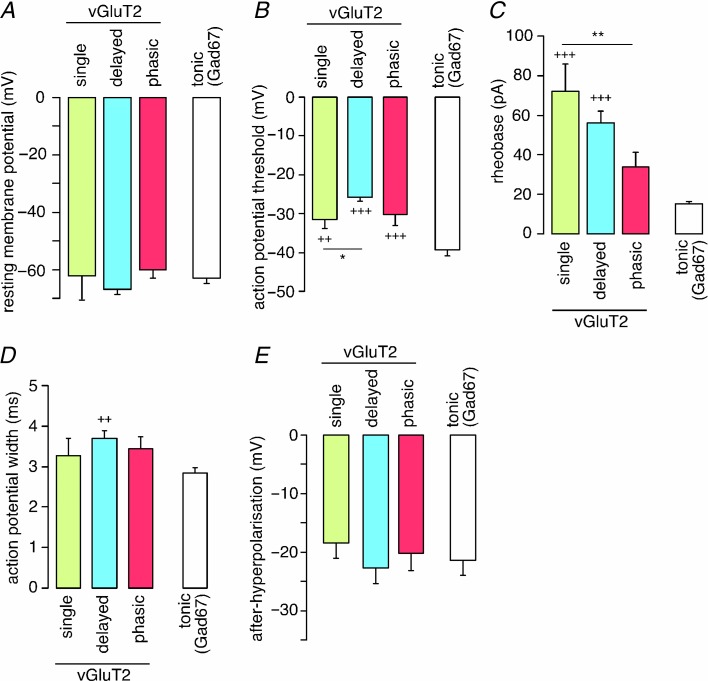
Different biophysical properties (*A*, resting membrane potential; *b*, action potential threshold; *C*, rheobase; *D*, action potential width; *E*, after-hyperpolarisation; values are means ± SEM) of three types of vGluT2::eGFP-positive neurons distinguished by their firing patterns (single, *n* = 9; delayed, *n* = 23; or phasic, *n* = 8) and of tonically firing Gad67::eGFP neurons (*n* = 32). **P < 0.01. *P < 0.05, significant difference between types of vGluT2::eGFP neurons. +++*P* < 0.001; ++*P* < 0.01, significantly different from Gad67::eGFP neurons. ANOVA followed by Bonferroni *post hoc* test. *F*(3,69) = 1.58 (resting membrane potential); 29.4 (action potential threshold; 22.1 (rheobase); 4.28 (action potential width); 0.87 (after-hyperpolarisation).

In a total of 42 neurons, we also tested the presence of direct or indirect synaptic input from primary nociceptive fibres. To this end, we perfused the slices with capsaicin (1 *μ*m), which excites primary nociceptors through activation of transient receptor potential vanilloid type 1 (TRPV1) channels, and tested whether this would increase the frequency of spontaneous postsynaptic currents (Fig. 6*A*). As expected from the preferential innervation of the superficial dorsal horn by primary nociceptors, an increase in EPSC frequency was more frequent in the superficial dorsal horn (14 of 21 neurons) than in the deep dorsal horn (7 of 21 neurons) (*χ*^2^(1) = 4.7; *P* < 0.05). When the prevalence of capsaicin-sensitive input was compared between vGluT2::eGFP, Gad67::eGFP and GlyT2::eGFP neurons, no significant differences were observed (*χ*^2^(2) = 3.75; *P* = 0.15, and (*χ*^2^(2) = 1.7; *P* = 0.44, for the superficial and deep dorsal horn, respectively; Fig. 6*B*).

**Figure 6:**
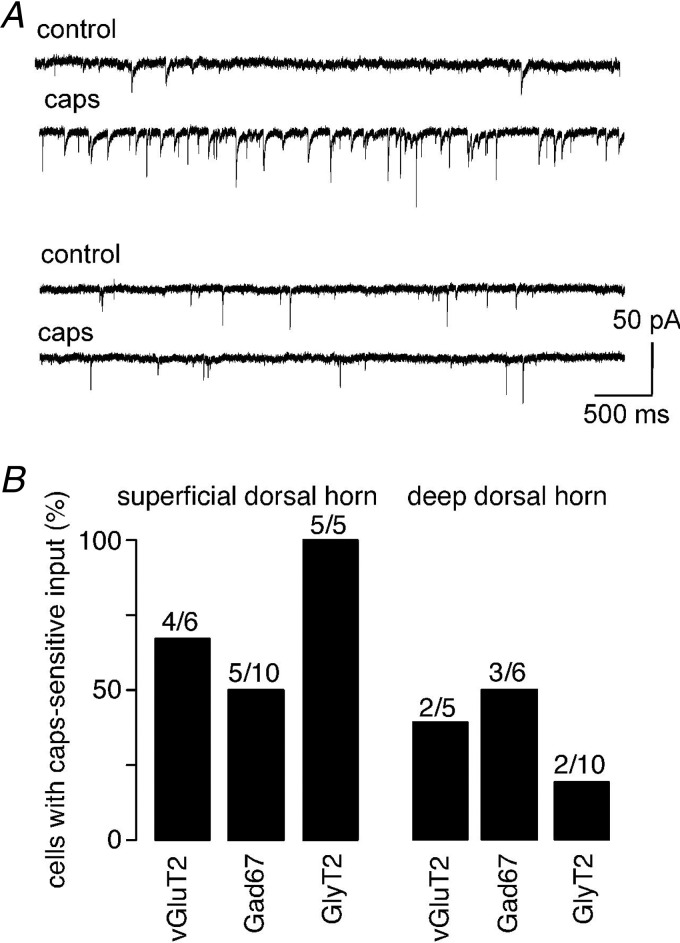
*A*, examples of cells receiving spontaneous capsaicin-sensitive synaptic input (top) or only capsaicin-insensitive synaptic input (bottom). *B*, portion of vGluT2::, Gad67::, and GlyT2::eGFP neurons receiving capsaicin-sensitive spontaneous synaptic input in the superficial and deep dorsal horn. Numbers of cells are indicated above the bars.

In a final set of experiments, we characterised the nature of the primary afferent-evoked (polysynaptic) inhibitory input onto the different eGFP-labelled neurons in the superficial dorsal horn (Fig. 7). Electrical dorsal root stimulation evoked excitatory synaptic input in all 41 neurons recorded. The presence of inhibitory postsynaptic currents (IPSCs) was investigated after switching to a holding potential of 0 mV, which was close to the actual reversal potential of EPSCs. After a control recording period, bicuculline (10 *μ*m) and a mixture of bicuculline (10 *μ*m) and strychnine (0.5 *μ*m) were applied consecutively (Fig. 7*Aa* and *b*). In several cells, we verified the polysynaptic nature of the IPSCs and showed that IPSCs disappeared in the presence of glutamate receptor blockers (20 *μ*m NBQX/50 *μ*m APV). Although primary afferent stimulation evoked EPSCs in all recorded neurons, IPSCs were detected only in a subset of neurons, i.e. in 9 out of 11 vGluT2::eGFP neurons as well as in 6 of 14 Gad67::eGFP and 12 of 16 GlyT2::eGFP neurons (Fig. 7*B* and *C*). All IPSCs had a strychnine-sensitive component, but between 17% (1 of 6 IPSCs in Gad67::eGFP neurons) and 58% (7 of 12 IPSCs in GlyT2::eGFP neurons) of the IPSCs lacked a GABAergic (bicuculline-sensitive) component, suggesting that glycine is the predominant fast synaptic inhibitor in all three neuron populations. A similar dominance of glycinergic inhibition was also present for the relative contribution of GABA and glycine to mixed IPSCs (Fig. 7*A–C*).

**Figure 7:**
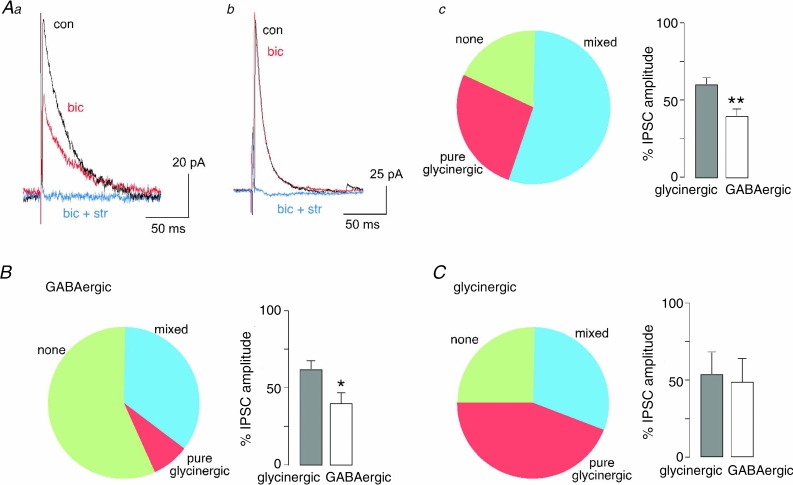
*A*, glutamatergic (vGluT2::eGFP) neurons recorded from the superficial dorsal horn (lamina I–II). *Aa*, example of a vGluT2::eGFP neuron receiving GABAergic and glycinergic input. Polysynaptic IPSCs were evoked by electrical stimulation of the dorsal root at C fibre strength (≥15 V). IPSCs were recorded under control conditions, in the presence of bicuculline (bic, 10 *μ*m), and in the combined presence of bicuculline and strychnine (bic + str, 10 *μ*m and 0.5 *μ*m, respectively). *Ab*, same as *Aa*, but example of a vGluT2::eGFP neuron with pure glycinergic inhibitory input. *Ac*, portion of vGluT2::eGFP neurons receiving both GABAergic and glycinergic inhibitory input, only glycinergic input, or no inhibitory input at all. Bar chart indicates relative GABAergic and glycinergic contribution to the total IPSC in neurons receiving mixed inhibitory input. Total number of neurons, *n* = 11. *B* and *C*, GABAergic, glycinergic neurons, same as *Ac*, but for Gad67::eGFP (*n* = 14) and GlyT2::eGFP neurons (*n* = 16), respectively.

## Discussion

The present study had two major aims. It was undertaken to characterise the eGFP expression in dorsal horn neurons of a vGluT2::eGFP mouse, which was generated by the Gensat Project and which is generally available for research. Secondly, we used this mouse line together with two other eGFP reporter lines (Gad67::eGFP and GlyT2::eGFP) expressing eGFP in two types of inhibitory neurons to perform a comparison of biophysical and physiological properties of excitatory and inhibitory neurons of the mouse dorsal horn.

### eGFP expression in dorsal horns of the vGluT2::eGFP transgenic mouse

The presence of at least one of the three vesicular glutamate transporter isoforms is an essential prerequisite for neurons to acquire a glutamatergic phenotype. The vast majority of spinal glutamatergic neurons express the isoform 2 of vGluT types (vGluT2; Oliveira *et al*. 2003; Todd *et al*. 2003; Alvarez *et al*. 2004; Malet *et al*. 2013). vGluT2 should therefore be well-suited as a marker gene for dorsal horn glutamatergic neurons. In order to verify, on a quantitative basis, the eutropic expression of eGFP in the spinal dorsal horn of vGluT2::eGFP mice, we stained spinal cord sections against the pan-neuronal marker NeuN and the transcription factor Pax2. As demonstrated in the present study, Pax2 is detected in more than 90% of all inhibitory (GABAergic or glycinergic) neurons of the spinal dorsal horn but is virtually absent in excitatory neurons. Because it is well established that nearly all non-inhibitory dorsal horn neurons are glutamatergic (Todd *et al*. 2003), we could thus identify excitatory neurons by the expression of NeuN in the absence of Pax2, whereas inhibitory neurons were positive for NeuN and Pax2. Our analyses confirmed the absence of vGluT2::eGFP from Pax2-positive (inhibitory) neurons. However, contrary to our expectations, only one-third of all Pax2-negative–NeuN-positive neurons expressed detectable levels of eGFP (even when eGFP detection was facilitated with anti-eGFP antibodies). Furthermore, the density of vGluT2::eGFP neurons showed a clearly visible dorsal–ventral gradient, which had not been detected in previous *in situ* hybridisation studies (Oliveira *et al*. 2003; Malet *et al*. 2013). Different factors need to be considered that may underlie the lack of expression of vGluT2::eGFP in the majority of dorsal horn excitatory neurons. eGFP-negative neurons might use vGluT isoforms different from vGluT2. However, vGluT1 is expressed only by a small number of dorsal horn neurons located in the intermediate zone and the dorsal nucleus of Clarke (Malet *et al*. 2013), and vGluT3 is only present in a rather small subpopulation of neurons in the deep dorsal horn (Malet *et al*. 2013). Conversely, previous *in situ* hybridisation experiments had revealed a wide-spread and strong vGluT2 labelling throughout the grey matter of the mouse spinal cord including the deep dorsal horn and in the ventral horn (Malet *et al*. 2013). Alternatively, vGluT2::eGFP might be detectable only in a fraction of vGluT2 neurons. This could either come from a low expression level not reaching the detection threshold, or from a restriction of eGFP expression to one or more subtypes of vGluT2 neurons. Analysis of co-expression of vGluT2::eGFP with established markers of subtypes of dorsal horn glutamatergic neurons (calbindin D-28k, c-Maf, NK1 receptor, PKC*γ*) did not support this latter possibility. Instead, all four markers were expressed in subsets of vGluT2::eGFP neurons, suggesting that the eGFP expressing neurons in the mouse line analysed here constitute a representative fraction of dorsal horn glutamatergic neurons. The alternative possibility that vGluT2::eGFP cells form a distinct population of excitatory neurons defined by an unknown yet-to-be-identified marker cannot, however, be fully ruled out.

Based on their high number and their location in the dorsal horn, most of the vGluT2::eGFP neurons are local excitatory interneurons. However, about 3% of all vGluT2::eGFP-positive neurons expressed NK1 receptors and all these neurons were located in the superficial dorsal horn. These neurons are most likely to be projection neurons (Marshall *et al*. 1996; Todd *et al*. 2000). About 4% of all vGluT2::eGFP neurons expressed PKC*γ*. Most of these were located in lamina IIi and resemble local excitatory interneurons. The majority of calbindin D-28k-positive and c-Maf-positive neurons probably are local excitatory interneurons but some of these neurons may also be projection neurons.

Most previous data on the expression of calbindin D-28k, NK1 receptor, and PKC*γ* in glutamatergic neurons of the dorsal horn have been obtained in rat (e.g. Yamamoto *et al*. 1989; Antal *et al*. 1991; Littlewood *et al*. 1995; Polgár *et al*. 1999). The new data presented here indicate that these proteins are also expressed in excitatory dorsal horn neurons of mice. Data allowing a quantitative comparison between rat and mouse is available for calbindin D-28k from Antal *et al*. (1991). This study found calbindin D-28k to be expressed in 16.4% of all rat excitatory lamina II neurons, which is in good agreement with the number reported in the present study for mice (14.9%).

Several previous studies have classified lamina II dorsal horn neurons according to the shapes of their primary dendritic trees. Most studies distinguished four types, namely vertical, radial, central and islet cells. These four classes (plus a fifth class termed ‘medial-lateral’) have originally been established for hamster superficial dorsal horn neurons (Grudt & Perl, 2002), but have been successfully applied since then also to rat and mouse (e.g. Heinke *et al*. 2004; Hantman *et al*. 2004; Maxwell *et al*. 2007; Yasaka *et al*. 2007, 2010). In our sample of 27 vGluT2::eGFP neurons recorded in parasagittal slices and filled with biocytin, about equal numbers of neurons showed characteristics of vertical, radial, and central cells. None of the 27 cells showed characteristics of islet cells (i.e. cells with a very long dendritic tree extending primarily in the rostro-caudal direction and with only short dorso-ventral and medio-lateral extensions). These results are in good agreement with previous reports that have proposed that virtually all islet cells are GABAergic (Gobel, 1975, 1978; Barber *et al*. 1982; Todd & McKenzie, 1989; Lu & Perl, 2003; Maxwell *et al*. 2007; Yasaka *et al*. 2010). In good agreement with our findings are also the reports by Yasaka *et al*. (2010), who found that vertical cells account for about one-third of all excitatory neurons in lamina II, and by Todd & McKenzie (1989), who showed that all vertical cells were non-GABAergic. Most previous investigators also found excitatory neurons in the populations of central cells (Grudt & Perl, 2002) and radial cells (Maxwell *et al*. 2007; Yasaka *et al*. 2010). Finally, in our study about one-fourth (7 of 27) of vGluT2::eGFP neurons had dendritic tree morphologies, which could not be attributed to one of the four classes. Similar portions of unclassified cells have been reported in studies by others (Grudt & Perl, 2002; Heinke *et al*. 2004; Maxwell *et al*. 2007; Yasaka *et al*. 2007, 2010). The present and the previous reports found that their dendritic trees were highly diverse and thus unlikely to define an additional class of neurons.

Taken together, the morphological data discussed above clearly demonstrate that eGFP expression in the dorsal horn of the vGluT2::eGFP BAC transgenic mouse studied here is highly specific for excitatory neurons. Previous studies that used reporter mice to specifically assess excitatory dorsal horn neurons had to take an indirect approach to identify excitatory neurons relying on the absence of reporter proteins driven by Gad65, Gad67 or GlyT2. Such a strategy is, however, not optimal given that none of these markers labels the whole population of inhibitory neurons. By contrast, in the vGluT2::eGFP mice studied here, eGFP was expressed in a subset of neurons likely to be representative of the whole population of excitatory dorsal horn neurons. This approach therefore allows the reliable identification of dorsal horn excitatory neurons making the vGluT2::eGFP mouse an ideal tool for the targeted recordings of excitatory dorsal horn neurons discussed below.

### Biophysical properties of vGluT2::eGFP neurons

vGluT2::eGFP neurons differed in several biophysical and physiological aspects from their inhibitory (GABAergic and glycinergic) counterparts. Most strikingly, vGluT2::eGFP neurons fired action potentials only at membrane potentials that were between 5 and 12 mV more depolarised than those of glycinergic and GABAergic interneurons. By contrast, no significant differences were found for the resting membrane potential, the cell capacitance, and the input resistance of the three cell types. Together, these four parameters explain why vGluT2::eGFP neurons required significantly stronger current injections to trigger action potentials than Gad67::eGFP and GlyT2::eGFP. If this difference applies also to physiological synaptic currents, it would indicate that excitatory neurons require stronger synaptic input than inhibitory interneurons to fire action potentials.

The analyses of action potential firing patterns in the different types of neurons show that excitatory neurons were not only more reluctant to fire action potentials but also fired fewer action potentials during prolonged depolarisations. Most vGluT2::eGFP neurons in the deep dorsal horn fired only single action potentials and about two-thirds of those in the superficial dorsal horn showed delayed action potential firing. None of the vGluT2::eGFP neurons recorded either in the superficial or deep dorsal horn showed tonic firing. By contrast, tonic action potential firing was by far the most prevalent firing pattern in inhibitory dorsal horn neurons of the superficial dorsal horn and the single most frequent firing pattern in deep dorsal horn inhibitory neurons. It therefore appears that in the superficial dorsal horn a tonic firing pattern is highly predictive for an inhibitory phenotype, while all other firing patterns are suggestive for an excitatory neuron. This correlation is weaker in the deep dorsal horn, where firing patterns differed from those in the superficial dorsal horn in at least two respects. Excitatory neurons in the deep dorsal horn showed more often a single action potential firing pattern than delayed firing. Second, firing patterns different from tonic firing were more frequently observed in inhibitory neurons in the deep dorsal horn than in superficial dorsal horn. In this context, it is interesting to note that the prevalence of the different firing types did not differ between transverse and parasagittal slices. The prevalence rates of the different firing patterns, in particular the high prevalence of tonic firing in GABAergic neurons reported here, are in good agreement with previous reports by Lu & Perl (2003), who described tonic firing as characteristic of rat lamina II islet cells, by Hantman *et al*. (2004), who found tonic firing in all (28) lamina II GABAergic neurons expressing GFP driven by the mouse prion promoter, and by Yasaka *et al*. (2010), who found a tonic firing pattern in 20/23 inhibitory lamina II neurons. Two other studies (Heinke *et al*. 2004; Hu & Gereau, 2011) reported lower prevalence rates of tonic firing in GABAergic dorsal horn neurons. The underlying causes of these differences are at present unknown, but they may come from the use of different transgenic mouse lines. Hu & Gereau (2011) and Heinke *et al*. (2004) employed the so called GIN mouse (Oliva *et al*. 2000), which carries a classical transgene driving expression of eGFP in about 35% of all GABAergic dorsal horn neurons, while the present study used the Gad67::eGFP which carries a targeted (‘knock-in’) construct driving eGFP expression in virtually all Gad67-positive neurons throughout the CNS (Tamamaki *et al*. 2003). While the present data together with previous reports by Lu & Perl (2003), Hantman *et al*. (2004), and Yasaka *et al*. (2010) suggest that the different firing patterns in dorsal horn neurons correlate with excitatory and inhibitory phenotypes, the results by Hu & Gereau (2011) and Heinke *et al*. (2004) call for caution. Both present and previous data indicate that the reliability of firing patterns as predictors of an excitatory or inhibitory phenotype does not reach that of genetically encoded reporters.

While there were clear differences between excitatory and inhibitory neurons in their biophysical properties and in their action potential firing patterns, only rather small differences were found between different subtypes of excitatory and inhibitory neurons. One exception however was the rheobase, which was highest in single spiking neurons and lowest in phasic firing vGluT2::eGFP neurons. We also found some differences between GABAergic and glycinergic interneurons. Gad67::eGFP neurons had more hyperpolarised action potential thresholds than GlyT2::eGFP neurons, and GlyT2::eGFP neurons had shorter action potentials than Gad67::eGFP neurons. Nevertheless the difference between these two inhibitory populations was smaller than that between inhibitory and excitatory neurons. This is consistent with previous reports that showed a large overlap of the GABAergic and glycinergic dorsal horn populations in particular in the deep dorsal horn (Mackie *et al*. 2003), where almost all glycinergic neurons also express markers of a GABAergic phenotype.

### Synaptic integration of different eGFP neurons

All of the three different types of dorsal horn neurons studied here received monosynaptic and/or polysynaptic input (i.e. via one or more intercalated excitatory interneurons) from capsaicin-sensitive (presumed nociceptive) primary afferents. As expected, neurons with nociceptive input were more abundant in the superficial than in the deep dorsal horn. We did not find statistically significant differences in the presence of nociceptive input between excitatory or inhibitory dorsal horn neurons (see also Heinke *et al*. 2004). While it is conceivable that the primary physiological function of input from capsaicin-sensitive fibres to excitatory dorsal horn neurons is the relay of nociceptive signals to the CNS, the physiological function of nociceptive input onto inhibitory neurons is less clear. It may contribute to nociceptive processing through feed-forward, feedback and lateral inhibition. Feed-forward inhibition of dorsal horn nociceptive relay neurons has been shown to limit nociceptive output of the spinal cord to the CNS (Baba *et al*. 2003; Torsney & MacDermott, 2006), while feedback inhibition may limit nociceptive input to the dorsal horn through presynaptic inhibition of primary nociceptor terminals (Rudomin & Schmidt, 1999; Willis, 1999). During acute pain, nociceptive input onto inhibitory neurons may contribute to ‘lateral inhibition’ and may thus help localising nociceptive stimuli to precise sites. Recent evidence in addition suggests that connections between primary nociceptive afferents and dorsal horn inhibitory neurons undergo a form of long-term depression, which may contribute to abnormal pain sensations in neuropathic pain states (Kim *et al*. 2012; Lu *et al*. 2013).

The presence of excitatory input from nociceptive fibres onto inhibitory dorsal horn neurons constitutes the basis for the primary afferent driven polysynaptic inhibitory input onto the dorsal horn described previously (Lu & Perl, 2003; Hantman *et al*. 2004; Heinke *et al*. 2004; Daniele & MacDermott, 2009). Here, we have characterised this polysynaptic inhibitory input in more detail. We found that glutamatergic neurons and both types of inhibitory neurons received polysynaptic primary afferent driven inhibitory input. In most of these cells, this inhibitory input was mixed GABAergic and glycinergic. However, in each of the three populations we also found cells that received only glycinergic input, whereas none of the cells received pure GABAergic inhibitory input. In cells with mixed inhibitory input, the contribution of glycine to the IPSC amplitude was bigger than that of GABA. This result was somewhat unexpected because the majority of inhibitory interneurons in the superficial dorsal are purely GABAergic (compare also panels *Bb* and *c* of Fig. 1 in the present report). On the other hand, previous studies from our group and from others had already shown that glycinergic IPSCs of superficial dorsal horn neurons are typically of larger amplitude than GABAergic IPSCs (Chery & De Koninck, 1999; Ahmadi *et al*. 2002; Pernía-Andrade *et al*. 2009). In all three dorsal horn neuron populations, between 20 and 60% of the cells did not receive any detectable inhibitory input. This portion was biggest for Gad67::eGFP neurons in which this portion reached 60%. Some of these cells might have been devoid of inhibitory input because they had lost part of its dendritic tree in the transverse slice preparation. However, we only included cells into this analysis that exhibited excitatory synaptic input after primary afferent stimulation, rendering a preparation artefact unlikely. In line with this conclusion is a previous study, which found that between 40 and 60% of Gad67::eGFP neurons of the outer lamina II lacked GABA_A_ receptors (Paul *et al*. 2012). Although it cannot be excluded that some of these cells still carried glycine receptors, it is likely that these cells correspond to those cells in the present study that were devoid of primary afferent driven IPSCs.

### Conclusions and further implications

The morphological and physiological characterisation of the vGluT2::eGFP mouse analysed here verifies a highly specific expression of eGFP in excitatory dorsal horn neurons. Although the penetrance of eGFP expression is incomplete in this mouse, the eGFP-tagged population of dorsal horn excitatory neurons is likely to be representative of the whole population of dorsal horn excitatory neurons. The availability of this mouse line should therefore greatly foster the functional analysis of glutamatergic neurons in dorsal horn circuits. The present study already shows that intrinsic properties of excitatory and inhibitory dorsal horn interneurons differ significantly. Excitatory neurons are less readily excitable than their inhibitory counterparts and fire fewer action potentials upon prolonged depolarisation. These findings are consistent with the idea that the relay of nociceptive signals through the spinal cord is under strong control by inhibitory neurons acting as gate keepers of pain (Melzack & Wall, 1965).

## Key points

- Excitatory and inhibitory interneurons of the spinal dorsal horn are critically involved in normal sensory processing and in the generation of pathological pain, but their physiological properties, especially those of excitatory interneurons, are only incompletely characterised.
- Here, we identified a vGluT2::eGFP BAC transgenic mouse line in which enhanced green fluorescent protein (eGFP) is specifically expressed in a subset of neurons that are likely to be representative of the whole population of excitatory dorsal horn neurons.
- We compared the physiological properties of vGluT2::eGFP neurons with those of inhibitory neurons in Gad67::eGFP and GlyT2::eGFP transgenic mice: vGluT2::eGFP neurons required stronger depolarising currents than inhibitory neurons to fire action potentials and fired fewer action potentials during prolonged depolarisations.
- Both excitatory or inhibitory dorsal horn neurons received synaptic input from capsaicin-sensitive fibres and primary afferent fibre-evoked (polysynaptic) inhibitory input.
- These findings should contribute to a better mechanistic understanding of normal and pathological sensory processing in the spinal dorsal horn.

## Additional information

### Author's present address

P. Punnakkal: Molecular Medicine, Sree Chitra Tirunal Institute for Medical Sciences and Technology, Poojappura, Thiruvananthapuram 12, Kerala, India.

## Abbreviations

AP: action potential
APV: (2*R*)-amino-5-phosphonovaleric acid
BAC: bacterial artificial chromosome
CB: calbindin D-28k
*C*_cell_: cell capacitance
DAB: 3,3′-diaminobenzidine
DAPI: 4′,6-diamidino-2-phenylindole
eGFP: enhanced green fluorescent protein
Gad: glutamate decarboxylase
GlyT: glycine transporter
NBQX: 2,3-dihydroxy-6-nitro-7-sulfamoyl-benzo[f]quinoxaline-2,3-dione
NDS: normal donkey serum
NGS: normal goat serum
NK1: neurokinin 1
PKC: protein kinase C
P: postnatal day
PFA: paraformaldehyde
*R*_input_: input resistance
TRPV1: transient receptor potential vanilloid type 1
vGluT: vesicular glutamate transporter
*V*_rest_: resting membrane potential
